# Combination of long- and short-read sequencing fully resolves complex repeats of herpes simplex virus 2 strain MS complete genome

**DOI:** 10.1099/mgen.0.000586

**Published:** 2021-06-25

**Authors:** Alberto Domingo López-Muñoz, Alberto Rastrojo, Kai A. Kropp, Abel Viejo-Borbolla, Antonio Alcamí

**Affiliations:** ^1^​ Centro de Biología Molecular Severo Ochoa (Consejo Superior de Investigaciones Científicas and Universidad Autónoma de Madrid), Madrid, Spain; ^2^​ Institute of Virology, Hannover Medical School, Hannover, Germany; ^†^​ Present address: Cellular Biology Section, Laboratory of Viral Diseases, NIAID, NIH, Bethesda, MD, USA

**Keywords:** HSV-2 MS, HSV isomers, long- and short-read sequencing, viral genome assembly

## Abstract

Herpes simplex virus serotype 2 (HSV-2) is a ubiquitous human pathogen that causes recurrent genital infections and ulcerations. Many HSV-2 strains with different biological properties have been identified, but only the genomes of HSV-2 strains HG52, SD90e and 333 have been reported as complete and fully characterized sequences. We *de novo* assembled, annotated and manually curated the complete genome sequence of HSV-2 strain MS, a highly neurovirulent strain, originally isolated from a multiple sclerosis patient. We resolved both DNA ends, as well as the complex inverted repeats regions present in HSV genomes, usually undisclosed in previous published partial herpesvirus genomes, using long reads from Pacific Biosciences (PacBio) technology. Additionally, we identified isomeric genomes by determining the alternative relative orientation of unique fragments in the genome of the sequenced viral population. Illumina short-read sequencing was crucial to examine genetic variability, such as nucleotide polymorphisms, insertion/deletions and sequence determinants of strain-specific virulence factors. We used Illumina data to fix two disrupted open reading frames found in coding homopolymers after PacBio assembly. These results support the combination of long- and short-read sequencing technologies as a precise and effective approach for the accurate *de novo* assembly and curation of complex microbial genomes.

## Data Summary

1. Raw sequence reads from both Pacific Biosciences (PacBio) and Illumina technologies are available at the European Bioinformatics Institute (EMBL-EBI) as Bioproject ID PRJEB40042.
2. The genome sequence of HSV-2 strain MS has been deposited in GenBank under accession no. MK855052.
3. A full list of minor variants (MVs) corrected in the PacBio assembly is available in Table S1 (available in the online version of this article), and MVs found in the HSV-2 MS genome are listed in Table S2. A full detailed list of each alignment hit between HSV-2 strain MS vs HG52, and MS vs 333, can be found in Tables S3 and S4, respectively.

**Impact Statement**

High-throughput sequencing (HTS) technologies have become a powerful tool in research and medical laboratories for disclosing microbial genomes, and short-read sequencing from Illumina technology is the default HTS choice to study and characterize microbial organisms easily. However, this short-read technology has limitations in determining the sequence of repeated and high GC-rich regions and resolving the complexity of poorly described areas in microbial genomes. The Pacific Biosciences (PacBio) sequencing technology, generating long-read sequencing reads, is an effective approach for resolving the complexity of large repeat regions, even those with homopolymeric structures. For cases such as HSV genomes, where central repeat regions together are often longer than 16 kbp, long reads facilitate *de novo* assembly, generating single continuous contigs covering the complete viral genome. Long contigs usually contain the resolved repeated regions, which can be used to interpret the relative position of unique elements within the viral genome, identifying isomeric genomes naturally present in viral populations. Short reads from Illumina are instrumental in identifying variants, insertions/deletions and other alterations, since the average read quality tends to be higher than that generated with PacBio long reads. The combination of these two technologies contributes to a more accurate and deeper understanding and characterization of genomes from large DNA viruses.

## Introduction

Herpes simplex virus 2 (HSV-2) is a major human pathogen that causes recurrent genital ulcerations after reactivation from the sacral dorsal root ganglia [1]. HSV may also cause life-threatening diseases, including disseminated disease in the neonate and herpes simplex encephalitis [2–4]. This virus has an estimated global prevalence for adult populations ranging from 11.3 % in 2012 [5] to 13.2 % in 2016 [6], depending on socio-economic status and country. HSV-2 contains a large, linear double-stranded DNA (dsDNA) genome of approximately 155 kilobase pairs (kbp) and is divided into unique long (UL) and unique short (US) regions, which are flanked by inverted repeats. There are a large number of clinical isolates and varieties of HSV-2 strains, with different neuroinvasion and virulence properties [7]. Some of them have been laboratory-adapted and partially characterized after serial amplification in cell culture. Nonetheless, the number of complete full-length genomic sequences available from these large number of HSV-2 isolates and strains remains quite low, being insufficient for accurate evolutionary comparisons and genomic determinant studies of virulence.

Variability in HSV genome size is mainly caused by the presence of variable nucleotide repeats, including microsatellites and tandem repetitions. The characterization of the flanking inverted repeats at both unique regions has been difficult because previous sequencing technologies could not resolve their length and sequence composition with accurate resolution [8–10]. Data from Illumina and Sanger technologies were used to assemble partial HSV genomes by using a consensus reference genome as template [11], where the average coverage and read quality at the inverted repeats tended to be too low for *de novo* assembly. Only a very few HSV-2 complete genomic sequences are currently available: laboratory strain HG52 [12, 13] and clinical isolates such as SD90e [13], among others [14]. There are also numerous partial genome sequences containing poorly covered regions and gaps at the frequently unresolved repeated regions [14–18]. By using Illumina and Pacific Biosciences (PacBio) sequencing technologies in combination, we successfully reported the complete genomic sequence of the widely used laboratory strain HSV-2 333 [19].

Here we have determined the complete genome sequence of the relevant HSV-2 strain MS, a clinical isolate originally obtained at the Department of Pathology, University of Iceland (Reykjavik, Iceland), from the midbrain of a patient with a 27-year history of multiple sclerosis, back in the 1960s [20]. We used PacBio long reads to *de novo* assemble the complete viral genome, and this was later curated using Illumina reads. Then, we characterized the level of genetic variability contained in the sequenced viral population by using Illumina reads, generating a list of high-confidence minor variants (MVs) found across the newly assembled genome. We also identified and fully characterized the *a* sequence of this type 2 strain, which plays an important role in HSV genome replication and isomeric conformation [21].

We found HSV-2 isomers by identifying long reads that mapped the orientation of the unique fragments and both central repeat regions. It is well known that replication of the HSV genome produces concatemeric DNA molecules [22]. Homologous recombination events mediated by the inverted repeat regions potentially lead to four genome isomers, which differ in the relative orientations of their unique UL and US fragments [21, 23]. As far as we know, this is the first time that long-read technology data have been used to successfully identify isomeric genome confirmations in viral populations. This approach can easily be used to study viral replication and recombination events in large DNA virus by determining isomer frequency as well as the main rearrangements occurring into the repeated regions. Full sequencing of additional HSV strains will improve our understanding of strain-dependent virulence and neurotropism [24], providing relevant information for antiviral drug and vaccine design.

## Methods

### Cell lines and virus

Cercopithecus aethiops kidney epithelial Vero cells (ATCC CCL-81) were cultured in Dulbecco’s modified Eagle’s medium (DMEM) supplemented with 5 % (v/v) foetal bovine serum (FBS), 2 mM l-glutamine and antibiotics (75 µg ml^−1^ penicillin, 75 U ml^−1^ streptomycin and 25 µg ml^−1^ gentamycin). Vero cells were cultured at 37 °C in a CO_2_-buffered cell incubator and regularly tested for mycoplasma contaminations by standard PCR with oligos Myco_Fw (GGCGAATGGCTGAGTAACACG) and Myco_Rv (CGGATAACGCTTGCGACCTAT). HSV-2 strain MS viral stock was kindly provided by Soren Riis Paludan (Aarhus University, Denmark).

### Viral DNA preparation

Two confluent P150-cm^2^ plates of Vero cells were infected at a multiplicity of infection (m.o.i.) of 0.1 plaque-forming units/cell and the viral inoculum was removed after 2 h post-infection (p.i.). Then, cells were overlaid with 20 ml of fresh DMEM containing 2 % FBS, 2 mM l-glutamine and antibiotics and incubated at 37 °C. Cells and supernatant were collected when 90–100 % of cytopathic effect was apparent and cellular debris was concentrated by centrifugation at 300 ***g*** for 5 min. The supernatant was discarded and 0.5 ml of 10 mM Tris/HCl pH 8.8 was added to the pellet, followed by three freeze/thaw steps. The pellet was disrupted by passage through a 23G needle and 1 ml syringe 30 times, releasing the intracellular virus. After 10 min of centrifugation at 300 ***g***, the supernatant was collected. This was repeated twice. The supernatants were combined and treated with DNAse I (0,25 U µl^−1^, Roche), RNAse A (20 µg ml^−1^, Roche) and nuclease S7 (0,25 U µl^−1^, Roche) for 2 h at 37 °C to eliminate cellular DNA/RNA. Viral particles were then lysed using the Proteinase K-SDS (Promega) method and DNA was purified using phenol : chloroform : isoamyl alcohol (25 : 24 : 1, v/v) extraction. All the procedures were performed in sterile conditions. Viral DNA was quantified in a Nanodrop One spectrophotometer (Thermo Fisher Scientific), as well as by fluorometric quantitation with a PicoGreen device (Invitrogen). Contaminating DNA was checked as previously described [25, 26] by standard PCR against mycoplasma, prokaryotic 16S rRNA with oligos 16S_Fw (CCTACGGGNBGCASCAG) and 16S_Rv (GACTACNVGGGTATCTAATCC), and eukaryotic 18S rRNA with oligos 18S_Fw (GCCAGCAVCYGCGGTAAY) and 18S_Rv (CCGTCAATTHCTTYAART).

### Construction and sequencing of PacBio and Illumina libraries

A high-quality genomic DNA (3000 ng) was submitted to the Norwegian Sequencing Centre, University of Oslo (Norway). A PacBio library was prepared using the PacBio protocol for SMRTbell Libraries using PacBio Barcoded Adapters for Multiplex SMRT Sequencing. The DNA sample was fragmented to 15 kbp (following sequencing chemistry specifications) by using a Megaruptor device, and size-selected using 0.45X AMPure PB beads. The library was sequenced on a PacBio Sequel instrument using Sequel Polymerase v2.1, SMRT cell v2 LR and sequencing chemistry v2.1. This library was pooled with another three after binding of the polymerase, and sequenced on approximately half of a SMRT cell. The loading was performed by diffusion. Run movie time was 1200 min and pre-extension time was 240 min. The resulting raw reads (77 235, average length of 3668 bp) were demultiplexed using Barcoding pipeline on SMRT Link (v 6.0.0.47841, SMRT Link Analysis Services and GUI v6.0.0.47836) with 26 as minimum barcode score. Sequence data were uploaded to the European Nucleotide Archive (ENA) and are available under accession number ERS3367571. The library for Illumina sequencing was prepared using the KAPA library prep kit (Kapa Biosystems). DNA was fragmented in a Covaris ultrasonic instrument and sequenced on an Illumina MiSeq device as paired-end 2×250 bp reads. Sequence data were uploaded to the ENA and are available under accession number ERS3367567.

### Genomic assembly, annotation and curation

PacBio reads were *de novo* assembled using the HGAP v4 [27] pipeline on SMRT Link (v 6.0.0.47841, SMRT Link Analysis Services and GUI v6.0.0.47836) with default settings. Illumina reads were quality-filtered (QF) with PrinSeq v0.20.4 [28] and then mapped to the PacBio assembled contig using BWA-MEM v0.7.17 [29] with default settings. This alignment was optimized with Picard-Tools v2.18.25 (http://broadinstitute.github.io/picard) and GATK v3.5 [30, 31] for automated genome assembly improvement, and variant detection–repair using Pilon v1.23 [32] (see Table S1a). Annotated coding sequences (CDSs), non-coding RNAs and structural unique/repeat regions were manually extracted from HSV-2 strain HG52 (accession no. NC_001798) and HSV-2 strain 333 (accession no. LS480640). Then, those features were aligned by using blastn v2.8.1 [33] against the longest PacBio assembled contig. All detected features were manually reviewed and annotated, revealing two insertions disrupting annotated CDSs, which were manually fixed by using Illumina data alignment (see Table S1b). Tandem repeat regions were annotated by using Tandem Repeats Finder [34]. The final genome sequence of HSV-2 MS was deposited in GenBank under accession no. MK855052.

### Variant analysis

Illumina QF reads were aligned against the HSV-2 MS final genome sequence by using Bowtie 2 v2.3.4.1 [35], with restrictive settings for higher accuracy (i.e. --n-ceil L,0,0.02; --rdg 0,6; --rfg 0,6; --score-min L,0,–0.24). MVs, single-nucleotide polymorphisms (SNPs) and insertions/deletions (InDels) were obtained with VarScan v2.4.3 [36] using standard settings with coverage ≥20 and minimum variant frequency ≥1 %, and ignoring variants with >90 % supported on one strand. A detailed list of these variants can be found in Table S2.

### Identification of genome isomers

Both internal repeat long (IRL) and internal repeat short (IRS) clusters (118 491–134 669) from HSV-2 MS genome were defined as reference spots. The UL (9629–118 490) and US (134 670–148 974) regions were then individually flipped to forward and reverse relative orientation to generate the four possible genomic isomers: UL-forward(fw)/US-reverse(rv); UL-fw/US-fw; UL-rv/US-fw and UL-rv/US-rv. Then, PacBio filtered reads longer than 12 kbp were aligned against each theoretical genomic isomer using blastn with default parameters. To define the relative orientation of the UL segment, aligned reads were filtered by determining whether their starting location was upstream of UL1 or UL56 CDSs (according to each orientation) and their ending location was downstream of the IRL–IRS border. Also, the US relative orientation was determined by mapping reads whose starting locations were upstream of the IRL–IRS border and whose mapped ending locations were downstream of the US1 or US12 start codon (for each orientation). For consensual reasons to other published HSV genomes, UL and US regions were both finally annotated and represented in forward relative orientation in HSV-2 strain MS.

### Phylogenetic analysis

The complete genome sequences for HSV-2 strains HG52, 333 and SD90e (accession no. KF781518) were retrieved from GenBank. The partial genome sequences of HSV-2 strain 333 (accession no. KP192856), strain 1192 (accession no. KP334095), strain COH 3818 (accession no. KP334096), strain CtSF (accession no. KP334097), strain CtSF-R (accession no. KP334093) and strain GSC-56 (accession no. KP334094) [16] were also retrieved from GenBank. HSV-1 strain 17 (NC_001806) was used as an outgroup. These sequences were aligned including the complete genome sequence of HSV-2 strain MS by using ClustalW [37] from the mega X software package [38], with standard parameters. A maximum-likelihood tree was generated using mega X software, with the general time-reversible nucleotide substitution model with five gamma categories, 1000 bootstrap replicates and complete deletion of alignment gaps, giving a total of 135 754 positions in the data set.

### Homology comparisons of HSV-2 MS genomic features

HSV-2 MS central *a* sequence was used to search similar sequences in the Nucleotide collection (nr/nt) database, using blastn v2.11.0 with megablast and default settings. Positive hits of relevant HSV-2 complete (but not partial) genomic sequences were selected, and then the HSV-2 MS *a* sequence was individually aligned against each of them. The identified and annotated central *a* sequence of each selected HSV-2 genome was used for multiple sequence alignment with hierarchical clustering [39].

Annotated CDSs and noncoding RNA (ncRNA) from HSV-2 strain MS were separately compared against HSV-2 strain HG52 and HSV-2 strain 333 complete genomes using blastn with default settings, and then homology alignments were represented by Easyfig v2.2.2 [40]. A full detailed list of each alignment hit can be found in Tables S3 and S4 (in blastn output format 6).

## Results and discussion

### Genomic sequencing, assembly and annotation

The genome of the HSV-2 strain MS, a highly neurovirulent strain, was sequenced first with the PacBio technology. We obtained a total number of 1523339 subreads from PacBio sequencing, which were *de novo* assembled with HGAP4 in default mode, giving three contigs as a result, with the longest being 155 961 bp long. After a dot plot analysis with Gepard v1.40 [41], the two shorter contigs were compared against the longest one, revealing that they were included in the sequence of the longest contig as portions of the IRL (but not IRS) region. Therefore, they were considered as assembly artefacts and discarded. Then, all reads were mapped to the longest contig, where 635 652 reads (41.73 %) aligned, giving an average coverage of 6278× (Table 1). Detailed analysis of the 155 961 bp long contig showed that the UL coding region was assembled in reverse relative orientation when compared by blastn alignment with HSV-2 strains HG52 and 333. We performed further analysis to determine whether the four possible HSV-2 isomeric genomes were present in the sequenced viral population. We reversed the UL segment to forward relative orientation in the HSV-2 MS annotated genome to maintain a consensus with previously annotated HSV-2 genomes. We also found a duplicated *a* sequence at the 5′ end of the contig, partially overlapping with a direct repeat (DR) 1 (DR1) sequence of 15 bp before the terminal repeat long (TRL) segment. This DR1 flanked both ends at the unitary *a* sequence, defining its borders [21]. Only a single copy of this DR1, but not the rest of the *a* sequence, was found at the 3′ end, after the terminal repeat short (TRS) segment. The *a* sequence plays a key role in circularizing the HSV genome [42, 43], where recombination events may happen, losing a full copy of the *a* sequence or a border-flanked DR1. Moreover, the *a* sequence contains the cleavage site of concatemeric DNA [44, 45], which is necessary for processing of unit-length genomes. This involves asymmetric cleavage of the DR1 shared by two adjacent *a* sequences [21]. Sequencing of these recombination intermediaries or asymmetric processed unit-length genomes could produce reads that would result in artefactual genome assemblies. Based on this, we relocated and restored one full copy of the *a* sequence from the 5′ end into the 3′ end, duplicating one DR1 to maintain the *a* sequence structure, resulting in a contig that was 155 976 bp long.

**Table 1.** PacBio and Illumina sequencing statistics of HSV-2 strain MS

Sequencing method	Aligner	Ref. genome for alignment	No. of reads	No. of mapped reads to ref.	% mapped reads	Average coverage	Max. coverage
PacBio	BWA-MEM	155 961 bp contig	1 523 339	635 652	41.73 %	6278	10 383
Illumina	BWA-MEM	155 976 bp modified contig	4 576 708	3 693 866	80.71 %	3401	4475
PacBio	BWA-MEM	155 975 bp final contig (accession no. MK855052)	1 523 339	637282	41.83 %	6282	10 383
Illumina	Bowtie 2	MK855052	4 576 708	3 382 632	73.91 %	3274	4475

**Fig. 1.**
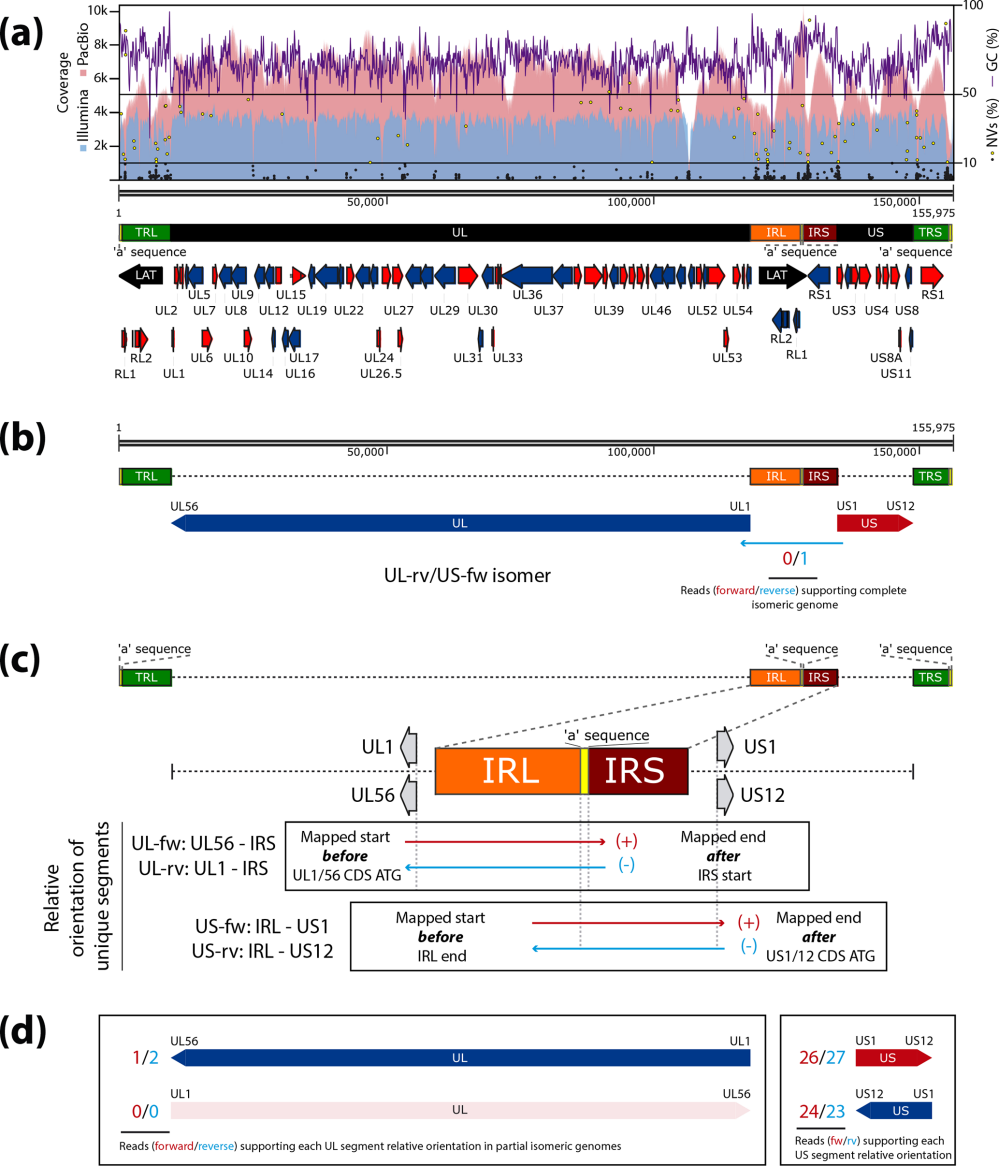
Genomic and genetic variability characterization of the annotated HSV-2 strain MS genome using PacBio and Illumina sequencing data. (**a**) Schematic of the HSV-2 strain MS genome. The orientation of each CDS is indicated in red (forward) and blue (reverse). Detected minor variants (MVs) are represented as black dots (frequency <10 %) and yellow dots (frequency >=10 %) in the graph. GC percentage plot (purple line), coverage plot from PacBio data alignment (red line) and coverage plot from Illumina data alignment (blue line) are mapped across the genome. (**b**) PacBio reads were aligned against each one of the four theoretical isomeric genomes. One long read was found, supporting the presence of the UL-rv/US-fw complete isomer in the sequenced viral population, mapping in reverse orientation (blue) across IRL and IRS regions and surpassing both *UL1* and *US1* CDS initiation codons. (**c**) PacBio reads were aligned against each one of the four theoretical isomeric genomes represented by alternative combinations of *UL1*, *UL56*, *US1* and *US12* flanked CDSs. The counting read approach was used to determine the relative orientation of each unique segment in partial genome isomers. Relative orientation of the UL segment was defined as UL-fw (forward oriented) or UL-rv (reverse oriented) by reads mapping before the initiation codon of *UL56* or *UL1* CDS, respectively, and after the IRS start. US segment, defined as US-fw or US-rv, by reads aligning before the IRL end and passing the initiation codon of *US1* or *US12* CDS, respectively. Mapped reads according to filtering parameters are indicated as forward (red) and reverse (blue). (**d**) Number of PacBio reads (in forward, red, and reverse, blue) found after alignment against each one of the four theoretical isomeric genomes, supporting the presence of each partial isomer regarding the relative orientation of UL or US segment.

Quality filtering of Illumina raw data provided 4 576 708 reads, of which 3 693 866 (80.71 %) mapped to the modified 155 976 bp long PacBio contig by BWA-MEM alignment, giving an average coverage of 3401× (Table 1). MV discrepancies in this optimized alignment were automatically detected and fixed (see Methods), with eight changes being made, as listed in Table S1a. Despite having done this, *UL10* and *UL36* CDS were found to be truncated due to a single nucleotide insertion, located in the homopolymeric regions. After detailed analysis of the frequency of these insertions in the optimized alignment, they were found to be represented with a very low penetrance in both cases (see Table S1b), and so they were manually fixed, giving a final contig that was 155 975 bp long. We then remapped the PacBio reads using BWA-MEM against the final contig, where 637 282 reads (41 83 %) mapped back and covered the whole genome with an average of 6282× (Table 1). We identified and annotated each feature, including CDSs, ncRNAs and poly-A signals (Fig. 1a), by blastn alignment of genomic features from HSV-2 strains HG52 and 333.

We found lower coverage in the large repeat regions than in the UL and US coding segments. The areas of lower coverage usually corresponded to high GC content (Fig. 1a). Long reads are highly indicated for *de novo* assembly of unknown genomes, since their length can accurately resolve complex repeat regions with high GC content [46]. PacBio reads are better at resolving homopolymeric regions, allowing the detection and characterization of DNA microsatellites, tandem repeats and their frequencies. However, the quality of reads tends to be lower than with short-read technology, which may result in biased genomic assemblies with disrupted open reading frames [47, 48], as also shown here. Short reads from Illumina technology provide data with higher quality, which is particularly useful for MV detection and InDel repair across *de novo* assembled genomes [11], proving to be critical for an accurate curation and annotation.

### Genetic variability: short-read variant analysis

Quality-filtered Illumina reads were realigned to the final genome sequence using Bowtie 2 with restrictive settings for higher accuracy (see Methods), with this aligner being better indicated for precisely accurate variant calling analysis than BWA-MEM [49]. After this, 3 382 632 reads (73.91 %) were mapped to the final genome, obtaining an average coverage of 3274× (Table 1). This final alignment was used to find MVs with a frequency greater than or equal to 1 %. To identify the genetic variability contained into the sequenced viral population, we plotted MVs across the HSV-2 MS genome to distinguish whether there were hotspots of variability, or whether it was evenly distributed (Fig. 1a). Terminal and internal inverted repeat regions showed the largest amount of variability, but this could be a biased result due to the lower coverage and higher GC % in some hot spots of these regions [11]. The analysis showed a general even distribution of variability across the UL and US regions, with the exception of few spots with a high degree of GC content as well as palindromic regions and, consequently, low coverage (Fig. 1a). We found a total number of 477 SNPs and 69 InDels with a wide range of frequency (see Table S2). Only 11 of these 69 InDels were found in CDSs, while 114 of 477 SNPs (~25 %) mapped in annotated CDSs, with 77 nonsynonymous SNPs. Among the latter, we identified two potential syncytial variants affecting the gene UL27 (MVs #131 and #132, Table S2), which encodes glycoprotein B, at amino acids 855 (R to H, 20.15 %) and 852 (A to V, 3.24 %), respectively. These two potential syncytia-inducing variants in UL27 are similar to some others previously described as affecting the same portion of the glycoprotein B tail region of HSV-1 [50–53]. It has recently been stated that the syncytial plaque phenotype conferred by these spontaneous mutations is favoured in Vero cells, dramatically increasing their frequency in the viral population after serial passage [54]. This is relevant, since it constitutes a clear example of how spontaneous mutations caused by genetic drift contribute to viral genomic adaptation to cell culture.

The significance of finding the majority of nucleotide polymorphisms in noncoding regions suggests that intergenic regions in HSV are genomic areas of low evolutionary constraint, where homopolymeric sequences, microsatellites and tandem repeats can easily vary with less selective pressure [16, 48, 55]. Using high-quality short reads has proven to be a powerful approach to uncovering the genetic variability present in this viral population. Further studies applying these approaches to HSV strain characterization will determine whether these intergenic regions play a fundamental role as variable regulatory regions behind differences in neurovirulence and neurotropism across HSV strains.

### Genomic variability: long-read isomer identification by UL/US relative orientation

In order to identify the genomic variability contained in the sequenced viral population (i.e. the four isomers of the HSV-2 MS genome), long-read alignments were used to find reads supporting the presence of each isomer. Both IRL and IRS, which separate UL and US regions, were selected as a reference cluster to define UL and US relative orientation through the presence of aligned filtered reads longer than 12 kbp (3579 reads in total), surpassing the borders. We only found a single read mapping across IRL and IRS regions and surpassing both *UL1* and *US1* CDS initiation codons at its ends, proving the presence of the UL-rv/US-fw isomer (Fig. 1b). This read would explain why the initial contig from PacBio data was assembled in the UL-rv/US-fw orientation. We did not find single reads confirming the presence of any other complete isomers, i.e. reads mapping from one unique region to the other, across the reference IRL–IRS cluster. Nonetheless, we found reads proving the presence of partial isomers, supported by reads resolving the relative orientation of one or another unique region, but not both at the same time (Fig. 1c). UL relative orientation was defined as UL-fw or UL-rv by reads whose mapping start aligned before the initiation codon of *UL56* or *UL1* CDS, respectively, and whose mapping end surpassed the IRS start. On the other hand, US relative orientation was defined as US-fw or US-rv by reads mapping before the IRL end and passing the initiation codon of *US1* or *US12* CDS, respectively.

We found 53 reads (26 fw+27 rv) supporting the US-fw relative orientation, mapping from IRL to *US1* CDS across the IRS (Fig. 1d). We also obtained 47 reads (24 fw+23 rv) showing the presence of the US-rv configuration (Fig. 1d), mapping from IRL to *US12* CDS across the IRS. In addition, we separately identified three reads (1 fw+2 rv) supporting the presence of the UL-rv segment, but no reads proving the existence of the UL-fw isomer (Fig. 1d). Although we could not directly determine the UL relative orientation in these partial isomers with a US-rv segment, we expect the presence of the UL-rv/US-rv genome isomer, since reads supporting both partial isomers separately were found. We also reanalyzed those two artefactual contigs generated after *de novo* assembly, in order to determine if there were isomer inversions of the main assembled contig. As commented previously, dot plot analysis performed with Gepard (data not shown) indicated that both short contigs were included in the longest assembled one, containing two copies of the *a* sequence, followed by IRL region and entering 5 and 12 kbp into the UL region, respectively. With this information, we were not able to determine if they were representatives of other isomeric forms in the viral population. It is likely for them to be replication intermediaries, as is suggested by the presence of two consecutive *a* sequences at one of the contig termini [45].

Despite not finding mapped reads, which would confirm the relative orientation of UL-fw and its two isomers, we annotated the HSV-2 MS genome with UL-fw/US-fw relative orientations to maintain consensual annotation with previously published strains. Size selection of 15 kbp fragments in the library preparation step and sequencing chemistry restrictions reduced the number of reads longer than 16 kbp, which is the length of the IRL–IRS cluster, and thus decreased the chance of finding reads supporting alternative isomeric genomes. Newer sequencing chemistries and instruments, which allow larger-insert libraries above 15 kbp, would be ideal in this regard. Moreover, a drop in PacBio coverage was found around the IRL–IRS border (see Fig. 1a), possibly due to a preferred physical breaking point in library preparation [8, 11]. Nonetheless, for HSV-1 strain KOS, it has been shown that specific isomers are overrepresented when cultured *in vitro* versus *in vivo* [22]. These reasons together could eventually explain why we did not find reads supporting the existence of the four isomers in the sequenced viral sample. However, our approach has proven to be a powerful tool to unravel the mechanisms behind genomic variability as a key component of viral evolution.

### Characterization of the *a* sequence

**Fig. 2.**
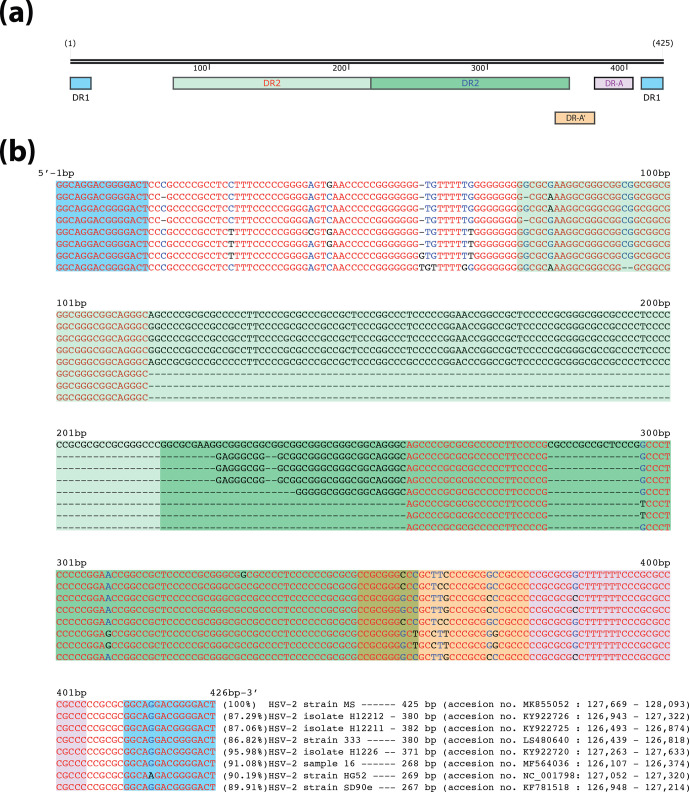
Characterization and homology comparison of the HSV-2 MS *a* sequence. (**a**) Schematic organization of the annotated *a* sequence from the HSV-2 MS genome containing direct repeat 1 (DR1), direct repeat 2 (DR2), direct repeat A (DR-A) and degenerated DR-A (DR-A’) sequences. (**b**) Multi-sequence alignment of the central inverted *a* sequence from complete genomic sequences of HSV-2 strains MS, 333, HG52, SD90e, isolates H12212, H12211, H1226 and sample 16. The percentage identity after blastn alignment between the HSV-2 MS *a* sequence and each strain/isolate is shown in parentheses, followed in each case by the length of the *a* sequence, the genome sequence accession number and the genomic coordinates used for this analysis. Each section of the multi-sequence alignment is highlighted by different colours according to the annotated direct repeats features in section (**a**). The consensus level of each position of the multi-sequence alignment is indicated as high (as red), low (blue) and neutral (black).

Two copies of the *a* sequence are present in the same orientation at the termini of HSV-2 genome and a third one is present in inverted orientation between the IRL and IRS regions. This sequence has an important role in efficient viral recombination and genome replication [56, 57]. It contains the packaging signal and the cleavage site of concatemeric DNA, where the alternative cleavage events promote, through recombination, the generation of the four genome isomers [22, 58]. We identified three *a* sequences within the HSV-2 strain MS genome, with each of them being 425 bp long. As represented in Fig. 2a, subsequent analysis showed a DR1 of 15 bp flanking both ends, two internal DR2 of 142 bp each, and two DR-A, with the first one (DR-A′) being more degenerated than the second DR-A. Previously described HSV-2 genomes may have partial or biased terminal *a* sequences [16–18], since they are located at the ends and flanked by TRL and TRS complex regions, and therefore they are difficult to sequence. Because of this, the internal inverted *a* sequence might be better identified, annotated and resolved, and would then be more suitable for strain comparison purposes. With this in mind, we searched for similar sequences to the HSV-2 MS central *a* sequence within the Nucleotide collection database by nucleotide megablast. Then, we selected the positive hits of relevant HSV-2 complete genomic sequences to individually align the HSV-2 MS *a* sequence against them, in order to identify and annotate their *a* sequences. The reference strain HSV-2 HG52 has an annotated internal *a* sequence of 254 bp, whereas that of the HSV-2 strain 333 is 380 bp long. Furthermore, every *a* sequence annotated in the HSV-2 HG52 genome has the same length, while the *a* sequences at the ends of the HSV-2 strain 333 genome are shorter than the one located between the IRL and IRS segments. The strain SD90e central *a* sequence is practically identical to that of strain HG52, since the consensus sequence of this strain was generated by using HG52 as the reference sequence for gap filling [13].

We identified a not previously annotated DR1 (genome coordinates 127 052–127 066), immediately upstream of the central *a* sequence in the reference strain HSV-2 HG52. We were able to successfully identify and annotate the central *a* sequence in all of these selected complete genomic sequences, based on the alignment of the HSV-2 MS central one (425 bp). These central *a* sequences, which exhibited a length ranging from 267 to 382 bp, were used for multiple sequence alignment analysis (Fig. 2b). Both flanking DR1s showed an identical structure and sequence across every strain/isolate, defining the beginning and end of every *a* sequence. Two-thirds of the first DR2 were completely missing in strains HG52, SD90e and sample 16, while some motifs of the second DR2 were highly conserved, but others were completely lost or highly degenerated. The first DR-A was found to show a high degree of variation, whereas the second exhibited high homology. These findings illustrated that the use of long-read data allowed us to identify, resolve and properly annotate both genomic termini, as well as the IRL–IRS border, in HSV-2 strain MS with unprecedented accuracy.

### Phylogenetic analysis of HSV-2 complete and partial genomes

**Fig. 3.**
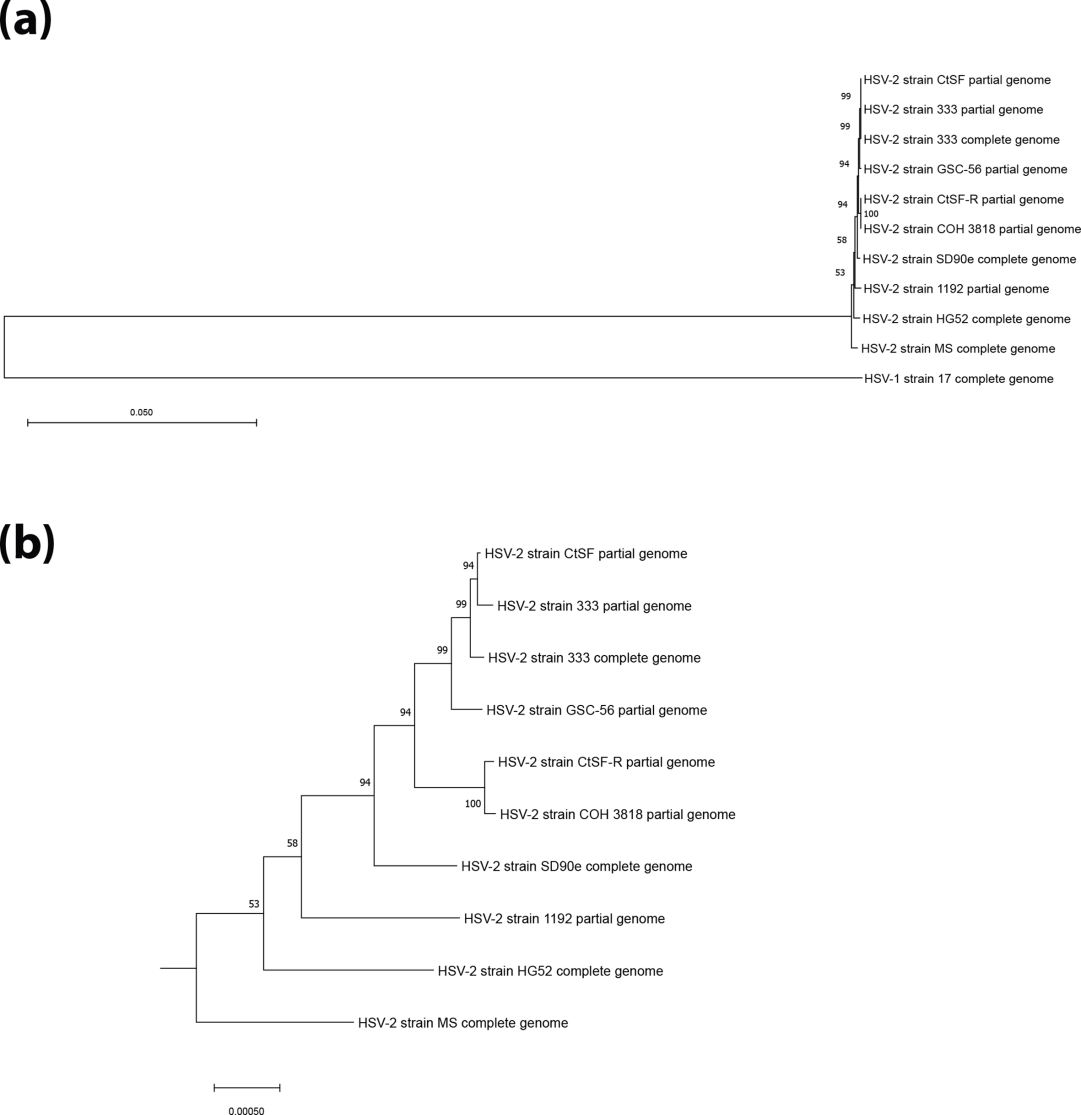
Phylogenetic tree of 10 HSV-2 complete and partial genome sequences, including the new HSV-2 strain MS. (**a**) Maximum-likelihood tree generated using the general time-reversible nucleotide substitution model with five gamma categories, 1000 bootstrap replicates and complete deletion of alignment gaps. HSV-1 strain 17 was used as outgroup. (**b**) Expansion of the HSV-2 cluster.

To investigate the relative phylogenetic position of the newly assembled HSV-2 strain MS genome within other complete and partial HSV-2 genomic sequences, we generated a maximum-likelihood-based tree including HSV-1 strain 17 (Fig. 3a), containing an expansion of the HSV-2 subtree (Fig. 3b). HSV-2 strains HG52 and 333 are high-passage-number laboratory strains, whereas SD90e, 1192, CtSF, CtSF-R, COH 3818 and GSC-56 are low-passage-number clinical isolates [16]. The phylogenetic tree data (Fig. 3a) clearly showed that HSV-2 and HSV-1 are separate species. Zooming into the HSV-2 node (Fig. 3b), the subtree showed the presence of clusters of strains CtSF, 333 (both partial and complete genomic sequences) and GSC-56 and of CtSF-R and COH 3818.

Despite being a complete genomic sequence, the strain SD90e was next found to be phylogenetically related to the previous node, followed by the more distant related strain 1192 (Fig. 3b). All of these previous strains and isolates seemed to be more phylogenetically distinct from strain MS than from strain HG52. Nonetheless, it must be taken into account that all of them, partial and complete genomes, were not *de novo* assembled, with the exception of the strain 333 complete genome [19]. They were generated using strain HG52 as the reference sequence for gap filling [13, 16], which is critically relevant in the repeat regions. The genomic information unresolved in those strains and filled up using the HG52 reference sequence could have contributed to their closer phylogenetic relationship to this strain than to the strain MS. As a relevant note, it is remarkable that the strain 333 partial genome was suggested to be phylogenetically closer to strain CtSF than to the complete version of itself. Together, these results highlight the critical relevance of the HSV inverted repeats, and their accuracy, when establishing phylogenetic relationships between herpesviruses.

### Whole-genome homology comparison of HSV-2 complete genomes

**Fig. 4.**
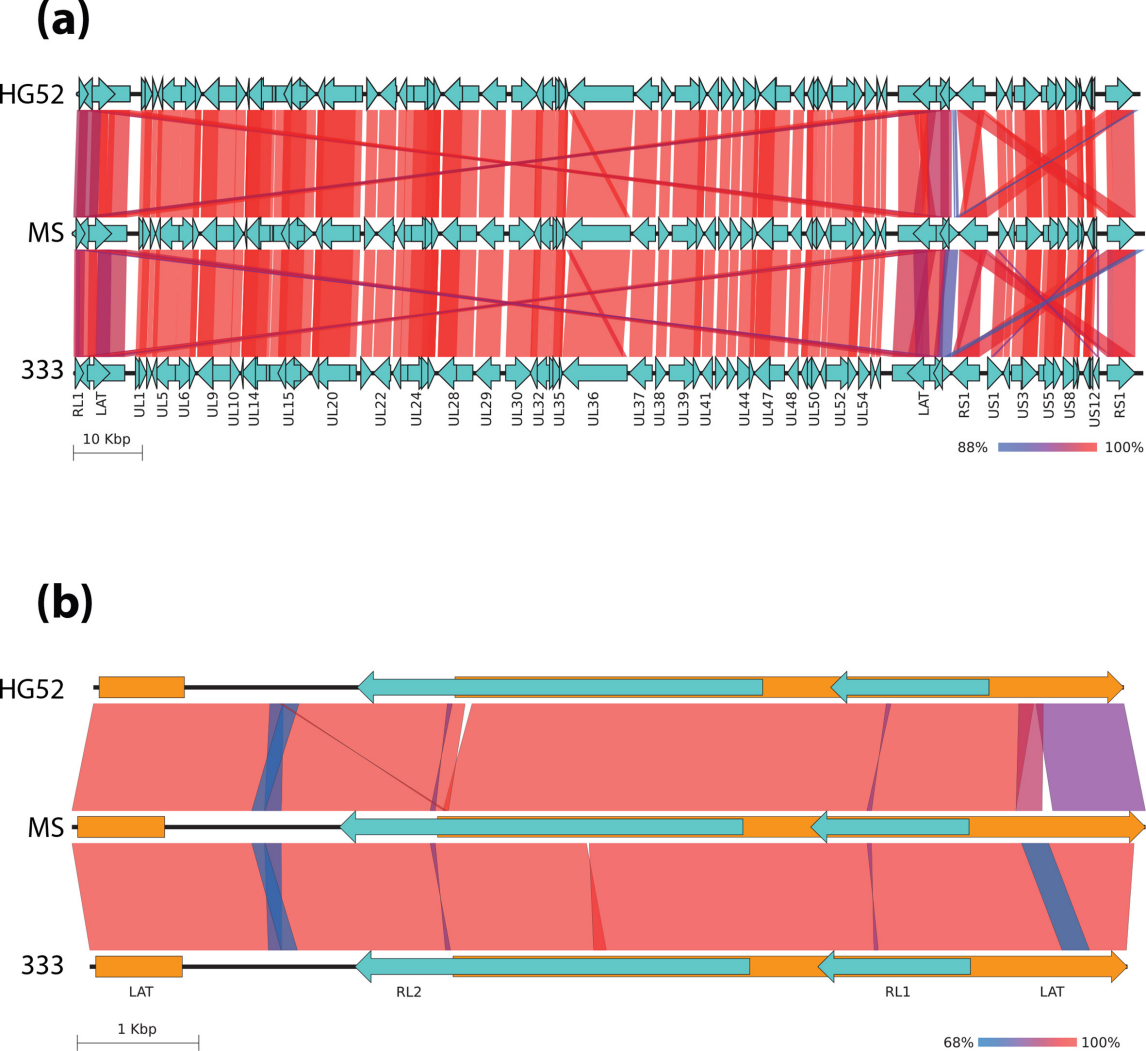
Genomic structure and homology comparison of HSV-2 complete genomes. (**a**) Homology alignment between CDSs and ncRNA from the genome of HSV-2 strain MS versus that of strain HG52 (upper pair), and strain HSV-2 MS versus strain 333 (lower pair), by blastn. (**b**) Homology alignments between central LAT ncRNA from HSV-2 strains MS and HG52 (upper pair) and strains HSV-2 MS and 333 (lower pair). Coloured scale bars indicate the percentage of homology, from completely identical (100 % in red) to the lowest value found for each case (in blue).

To compare the whole-genome homology, we selected the two *de novo*-assembled sequences that were most phylogenetically distinct from the HSV-2 strain MS, based on the previous phylogenetic analysis. The genomic structure and homology level of each annotated CDS and ncRNA were compared between the complete genomes of HSV-2 MS and HG52 and HSV-2 MS and 333, respectively, by blastn alignments of those genomic features (Fig. 4a, see also Tables S3 and S4). As expected, the homology between HSV-2 genomes was very high and widely conserved within the UL and US regions. CDSs were similar in relative locations across the genome, and showed high levels of homology. The lowest homology rates were observed in the inverted repeat regions, particularly between the ncRNA latency-associated transcript (LAT), with a homology rate of 88–90 % (Tables S3 and S4). Central LAT in the IRL–IRS border from the MS strain was longer (8 841 bp) than in the 333 (8 544 bp) and the HG52 strains (8 485 bp). By additional blastn alignments between LAT sequences from each HSV-2 complete genome, we found the lowest homology rates between areas around the LAT intronic region, and at the 3′ end, where the *a* sequence defines the IRL–IRS border (Fig. 4b). This low homology level was more noticeable when comparing the MS to the HG52 strain, than MS vs 333, since the HG52 genome was only assembled using short-read technologies [16, 17]. Therefore, the level of variation we found at this point (as well as others yet to be found) might be caused by differential passage history and adaptation in cell culture, but also by the intrinsic variation of Illumina sequencing when using this for high GC content and repeated clusters. These regions are particularly difficult to sequence, assemble and resolve, especially when only short-read technologies are used. Ultimately, it would be ideal to be able to sequence the original clinical isolate, prior to any propagation in cell culture, in order to assess the genomic differences between different strains.

## Conclusion

Long-read technologies provide data that are useful in determining the complete genomes of microbes, especially those that have large and complex repeat regions. As we report here, these data are useful in resolving both the sequence and the relative orientation of difficult regions with a high degree of accuracy, as well as the 5′ and 3′ terminal repeat regions from large DNA viruses. The long-read data are fundamental for precise and successful assembly, in order to define differences in genomic structure and homology between highly related viral strains [59, 60]. However, the data quality of these long-read technologies may be too low for successful identification of MVs, as well as the frequency of those across newly assembled genomes. Short-read technology, on the other hand, is very useful for variant detection and InDel curation, as shown here and previously [19]. However, this short-read technology is poor at determinining the length of repeat regions that are longer than the average read length, or the number of repeats. Together with the analysis of the genomic structures and homology between strains, variant analysis is decisive for a better understanding of differences in virulence, tropism and pathogenicity among HSV isolates [11, 24, 61, 62]. Thus, we propose that the combination of long- and short-read HTS technologies represents a powerful and accurate approach for *de novo* assembly of large and complex microbial genomes [46, 63], facilitating new insights into the sequence determinants of viral pathogenesis and serving as a tool for the future design of vaccines and antiviral drugs [64].

## Supplementary Data

Supplementary material 1
